# Osteopontin and MMP9: Associations with VEGF Expression/Secretion and Angiogenesis in PC3 Prostate Cancer Cells

**DOI:** 10.3390/cancers5020617

**Published:** 2013-05-27

**Authors:** Aditi Gupta, Cindy Q. Zhou, Meenakshi A. Chellaiah

**Affiliations:** Department of Oncology and Diagnostic Sciences, Dental School, University of Maryland, Baltimore, MD 21201, USA

**Keywords:** osteopontin, integrin αvβ3, MMP9, VEGF, angiogenesis, curcumin

## Abstract

Osteopontin and MMP9 are implicated in angiogenesis and cancer progression. The objective of this study is to gain insight into the molecular mechanisms underlying angiogenesis, and to elucidate the role of osteopontin in this process. We report here that osteopontin/αvβ3 signaling pathway which involves ERK1/2 phosphorylation regulates the expression of VEGF. An inhibitor to MEK or curcumin significantly suppressed the phosphorylation of ERK1/2 and expression of VEGF. MMP9 knockdown reduces the secretion but not the expression of VEGF. Moreover, MMP9 knockdown increases the release of angiostatin, a key protein that suppresses angiogenesis. Conditioned media from PC3 cells treated with curcumin or MEK inhibitor inhibited tube formation *in vitro* in human microvascular endothelial cells. Similar inhibitory effect on tube formation was found with conditioned media collected from PC3 cells expressing mutant-osteopontin at integrin-binding site and knockdown of osteopontin or MMP9. We conclude that MMP9 activation is associated with angiogenesis via regulation of secretion of VEGF and angiostatin in PC3 cells. Curcumin is thus a potential drug for cancer treatment because it demonstrated anti-angiogenic and anti-invasive properties.

## 1. Introduction

Prostate cancer is the second leading cancer that causes death in men and it is the most commonly diagnosed cancer in the U.S. It is a disease of extensive metastasis, with secondary lesions in the lymph node, brain, bones, and sometimes in visceral organs such as liver and lungs. Metastasis of malignant tumor cells is dependent on the formation of new blood vessels from existing ones, a process which is known as angiogenesis [1]. It is one of the fundamental processes required for solid tumor growth, survival and metastasis. A number of autocrine and paracrine factors facilitate angiogenesis. Vascular endothelial growth factor (VEGF) is known to be one of the most potent angiogenic factor due to its specificity to endothelial cells [2]. VEGF, a 34–42 kDa glycoprotein recognizes two major tyrosine kinase receptors (VEGF-R1 and -R2). 

VEGF expression has been found in prostate cells of normal, benign, and malignant types [3,4]. It plays a role in the microvascular remodeling in prostate cancer induced osteolysis and bone loss [5]. Of the four VEGF isoforms (VEGF-121, -165, -189, and -206) identified [6,7], VEGF-165 is predominantly implicated in physiological and pathological angiogenesis [8]. VEGF-165 henceforth denoted as VEGF. Overexpression of VEGF-165b by tumor cells inhibits the growth of prostate carcinoma. VEGF-165b is implicated as anti-angiogenic factor [9].

Osteopontin (OPN) produced by tumor cells has the potential to enhance the metastatic ability through regulation of VEGF secretion and angiogenesis [10,11]. Cooperative mechanisms involving osteopontin and αvβ3 are implicated in VEGF-mediated endothelial cell migration and angiogenesis. A monoclonal antibody (LM609) to αvβ3 suppressed angiogenesis [12,13]. These observations suggest OPN and VEGF as important regulators of angiogenesis through integrin αvβ3 signaling pathway. Curcumin modulates multiple pathways and several of its molecular targets relevant to prostate cancer have been identified [14]. In addition to their effects on proliferation, curcumin and inhibitors to MMPs and MEK were shown to abrogate pathological angiogenesis [15].

Studies on the function of active MMP9 reported that it triggers the angiogenic switch during carcinogenesis [15,16]. Upregulation of secreted MMP9 correlated with an increase in tumor growth and angiogenesis compared to cells expressing low MMP9 levels [17]. We have shown previously that OPN and αvβ3 signaling regulates the activity of MMP9 in prostate cancer PC3 cells. PC3 cells expressing mutated OPN at integrin-binding site [PC3/OPN (RGA)] and knockdown of OPN [PC3/OPN (KD)] displayed a significant decrease in MMP9 activity [18]. Our aim in this study is to identify the specific roles of OPN and MMP9 in VEGF-mediated angiogenesis using respective PC3 knockdown cells. In the present study we have shown that osteopontin/αvβ3 signaling which involves ERK1/2 phosphorylation regulates VEGF expression in PC3 cells. MMP9 activation is associated with angiogenesis via regulation of secretion of VEGF and inhibition of secretion of angiostatin by PC3 cells. 

## 2. Experimental Section

### 2.1. Materials

Antibodies to VEGF, GAPDH, and β-Actin as well as HRP-conjugated secondary antibodies (rabbit, goat and mice) were purchased from Santa Cruz Biotechnology, Inc. (Santa Cruz, CA, USA). An inhibitor to MEK1/2 inhibitor (U0126) and antibodies to phospho-ERK and ERK were purchased from Cell Signaling Technology, Inc. (Danvers, MA, USA). Polyvinyldifluoride (PVDF) membrane for immunoblotting analysis and Amicon centrifugal concentrator devices for concentrating the protein in the conditioned media were obtained from Millipore Corp. (Bedford, MA, USA). Human prostate tumor and normal tissue lysates (total tissue, membrane and nuclear lysates) were purchased from Abcam (Cambridge, MA, USA). Matrigel was purchased from BD Biosciences (Bedford, MA, USA). Curcumin [1,7-bis(4-hydroxy-3-methoxyphenyl)-1,6-heptadiene-3,5-dione] was purchased from Sigma Chemical Co. (St. Louis, MO, USA).

### 2.2. Cell Lines and Culture Conditions

*Cell lines.* Prostate cancer epithelial cell lines that stably express high levels of osteopontin (OPN; full length and mutant-RGDΔRGA) and knockdown of OPN (PC3/OPN (KD) or MMP9 (PC3/MMP9 (Si) were generated as described previously [18,19]. These clones were designated as PC3/OPN, PC3/OPN (RGA), PC3/OPN (KD) and PC3/MMP9 (Si). Normal prostatic epithelial cells (HPR-1) were used as controls [20]. These cell lines were cultured as described previously [18,19]. Human microvascular endothelial cells (HMEC-1) were used for angiogenesis assay *in vitro*. HMEC-1were cultured in MCDB-131 medium (Invitrogen, Carlsbad, CA, USA) supplemented with 10% fetal bovine serum (FBS), 10 mM L-glutamine (invitrogen 25030), 1 µg/mL hydrocortisone (Sigma), 10 ng/mL epidermal growth factor (BD) and 1% (v/v) penicillin-streptomycin at 37 °C, in a 5% CO_2_ humidified atmosphere.

### 2.3. Treatment of PC3 Cells with MEK Inhibitor and Curcumin

PC3 cells cultured in RPMI-1640 media containing 10% FBS at 37 °C were treated with thefollowing at the indicated time and concentration: 10 μM MEK inhibitor (U0126) for 48 h; curcumin 40 μM for 24 h. Following various treatments, lysates were made with cold RIPA lysis buffer as described previously [21]. 

### 2.4. Quantification of VEGF in the Conditioned Medium

Cells were grown to 80–90% confluence in RPMI-1640 medium containing 5% FBS. They were washed thrice with pre-warmed serum-free medium and incubated with the same for 72 h at 37 °C. Conditioned media were collected and concentrated approximately 10-fold with a Centricon concentrator (Amicon, Beverly, MA, USA) [18]. Quantification of the secreted VEGF in the conditioned media of PC3 cell lines was done by comparative analysis with different concentrations of either BSA or purified recombinant VEGF using 12% SDS-PAGE. Coomassie blue staining was done in gels loaded with BSA. Immunoblotting analysis was done with an antibody to VEGF in gels loaded with different concentrations of recombinant VEGF (R&D systems; Minneapolis, MN, USA). 

### 2.5. Tube Formation: An *in Vitro* Matrigel Angiogenesis Assay

Capillary tube formation assays were performed using HMEC-1 essentially as described [22]. Briefly, 200 μL thawed matrigel on ice was pipetted into pre-chilled 24-well plates and allowed to polymerize for 1 h at 37 °C. To investigate the response of HMEC-1 to different conditioned media of PC3 cells on *in vitro* angiogenesis, HMEC-1 were seeded (~1 × 10^5^ cells/mL) on the matrigel and cultured in MCDB-131 medium with equal amount of conditioned media protein from indicated PC3 cell lines in the results section. The plates were then incubated at 37 °C in a humidified atmosphere of 95% air and 5% CO_2_. Capillary tube formations on matrigel were visualized after 24 h under an inverted phase-contrast microscope. 

### 2.6. Immunoblotting and Gelatin Zymography Analyses

Equal amount of protein lysates were used for immunoblotting analyses as described previously [21]. Conditioned media protein was diluted and used for gelatin zymography analysis as described previously [18].

### 2.7. Immunohistochemistry

Prostatic adenocarcinoma tissue microarray (TMA) sections containing six cases of prostate adenocarcinoma with six adjacent normal prostate tissues in duplicate cores per case were purchased from the US Biomax, Inc. (Rockville, MD, USA). TMA sections were processed, stained with VEGF antibody, and analyzed essentially as described previously [23]. 

### 2.8. Statistical Analysis

All values are presented as mean ± SEM. A value of *p* < 0.05 was considered significant. Statistical significance was determined by analysis of variance (ANOVA) with the Bonferonni corrections (Instat for IBM; GraphPad software; San Diego, CA, USA). Statistically significant difference in VEGF distribution was determined between prostatic adenocarcinoma and normal prostatic epithelial cells as specified in Figure 7. 

## 3. Results

Vascular endothelial growth factor (VEGF) was shown to have an effect on the proliferation of prostate cancer cells found in the bone microenvironment [24]. Therefore, we have primarily used PC3 cells derived from bone metastasis. A positive correlation between OPN and VEGF concentrations was found in synovial fluid in synovial tissue [24]. To determine the contribution of OPN/αvβ3 signaling in the expression of VEGF and angiogenesis, we have used PC3/OPN (RGA) which demonstrated a decrease in integrin signaling [18]. We have also used PC3/OPN (KD) cells [18,25]. 

### 3.1. Regulation of VEGF Expression by Osteopontin

**Figure 1:**
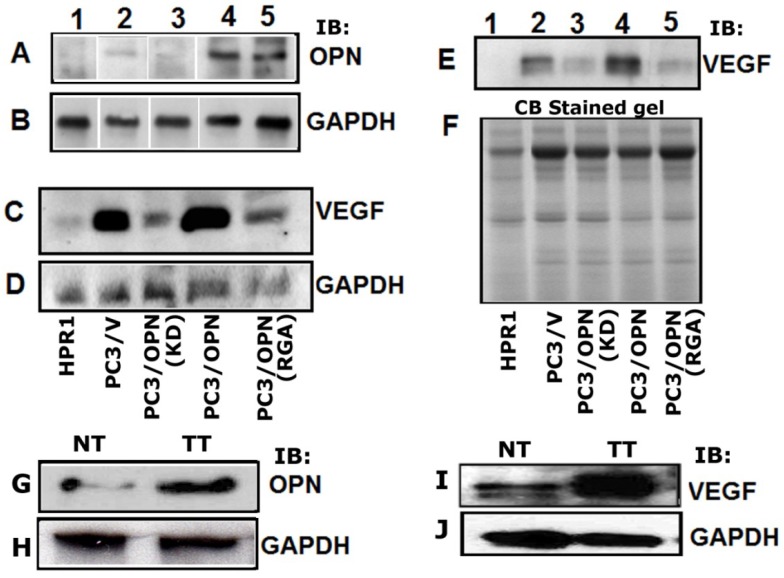
Osteopontin induces VEGF expression. Equal amount of protein from indicated PC3 cell lines was used for immunoblotting analysis with an antibody to OPN (**A**) and VEGF (**C**, **E**, and **G**). Total cellular lysate (**A** and **C**) and conditioned media (**E**) proteins (20µg protein) were used for this analysis. Lysates made form prostatic normal (NT) and tumor (TT) tissue were also used for immunoblotting analysis with an antibody to OPN (**G**) and VEGF (**I**). GAPDH was used as a loading control (**B**, **D**, **H** and **J**). The loading control for the conditioned media is shown by the use of Coomassie blue staining of a gel ran in parallel (**E**). The results shown are representative of three or four experiments.

As shown previously [18], OPN expression is considerably more in PC3/OPN and PC3/OPN (RGA) (Figure 1A, lanes 2 and 3) cells as compared with vector DNA transfected control PC3 cells (PC3/V; lane1). A significant decrease in OPN expression was observed in PC3/OPN (KD) (lane 4) and normal prostatic epithelial cells (HPR-1, lane 5). Subsequently, total cellular (Figure 1C) and secreted (Figure 1E) levels of VEGF were determined in these cell lines by immunoblotting analysis. A significant increase in the cellular (Figure 1C, lane 4) and secreted (Figure 1E, lane 4) levels of VEGF was observed in PC3/OPN cells as compared with control cells. However, a significant decrease below the level detected in PC3/V cells (C and E, lane 2) was observed in PC3/OPN (RGA) and PC3/OPN (KD) cells (C and E, lanes 3 and 5). 

VEGF expression is very minimal in HPR-1 cells (C and E, lane 1). The expression level was observed in the following order: PC3/OPN > PC3/V >>PC3/OPN (RGA) = PC3/OPN (KD) >> HPR-1. Immunoblotting analyses of lysates from prostate tumor tissue (TT; panel G) demonstrated that OPN and VEGF expression is significantly more in TT than normal tissue (NT, panel G and I). Taken together, these results indicate that OPN induces expression of VEGF in PC3 cells. Integrin signaling may play a role in this process because a decrease in VEGF expression was observed in PC3 cells expressing mutant OPN at integrin binding site (RGDΔRGA). These observations together with others demonstrate an increase in the expression of OPN and VEGF in cancer cells [26,27,28,29].

### 3.2. Osteopontin Deficiency Suppresses VEGF-Induced Angiogenesis *in Vitro*

**Figure 2:**
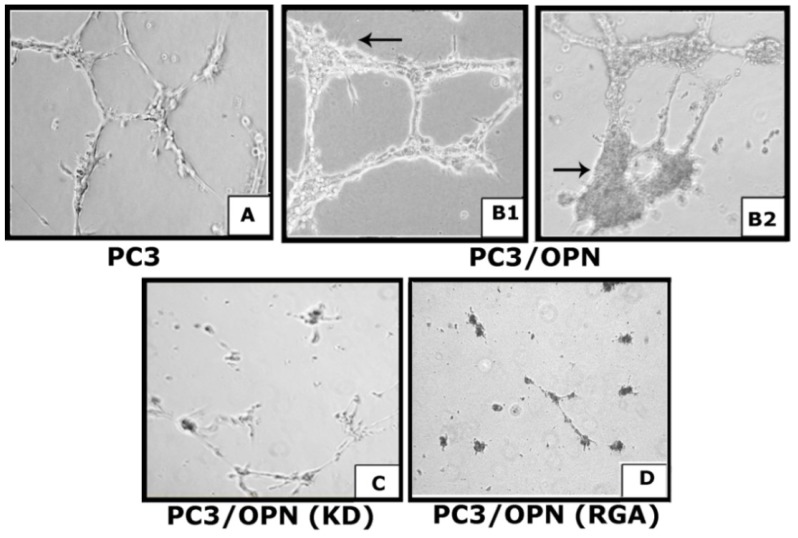
Secreted OPN and VEGF promote endothelial cell migration and microvascular tube formation *in vitro*. Phase contrast micrograph showing tube formation *in vitro* by HMEC-1 cells. HMEC-1 cells were grown on matrigel and incubated with conditioned media from indicated PC3 cells for 24 h. Images were captured (×200) with an inverted phase contrast microscope. Results shown are representative of three independent experiments.

Subsequently, we examined the efficacy of VEGF in the conditioned media of different PC3 cells to induce endothelial cell proliferation and formation of network of interconnecting tubules-like structures *in vitro* (Figure 2). HMEC-1 were used for this purpose. We found that conditioned media from PC3 and PC3/OPN cells induced the formation of tubular sprouts (Figure 2A,B). 

The tubular sprouts were thicker in endothelial cells incubated with the conditioned medium from PC3/OPN cells (B1 and B2). Furthermore, we observed clusters of cells at the intersections (corners) of intertubular networks (indicated by an arrow in B1 and B2). This suggests that OPN in the conditioned media of PC3/OPN cells not only stimulates proliferation but also adhesion by clustering of endothelial cells. Neither tube formation nor proliferation was observed with conditioned media from PC3/OPN (KD) and PC3/OPN (RGA) cells (C and D). Taken together, these data suggest that secretion of OPN and VEGF by PC3 cells can induce angiogenesis process in bone microenvironment. OPN not only can increase VEGF expression but also adhesion of cells which can possibly occur by interaction of OPN with cell surface adhesion molecules of HMEC-1 cells. 

A considerable number of reports have shown that curcumin has anti-cancerous and chemotherapeutic effects. A series of experiments have been done in PC3 cells to determine the effects of curcumin on VEGF expression, MMP9 activity, and VEGF secretion. To corroborate that reducing MMP9 activity by curcumin blocks VEGF secretion, we used PC3 cells knockdown of MMP9. We also determined whether curcumin and MAPK inhibitor has similar inhibitory effects on VEGF expression and there is any cross communication occur between the ERK pathway and VEGF expression.

### 3.3. Curcumin Down-Regulates Vascular Endothelial Growth Factor (VEGF) Expression in PC3 Cells

**Figure 3:**
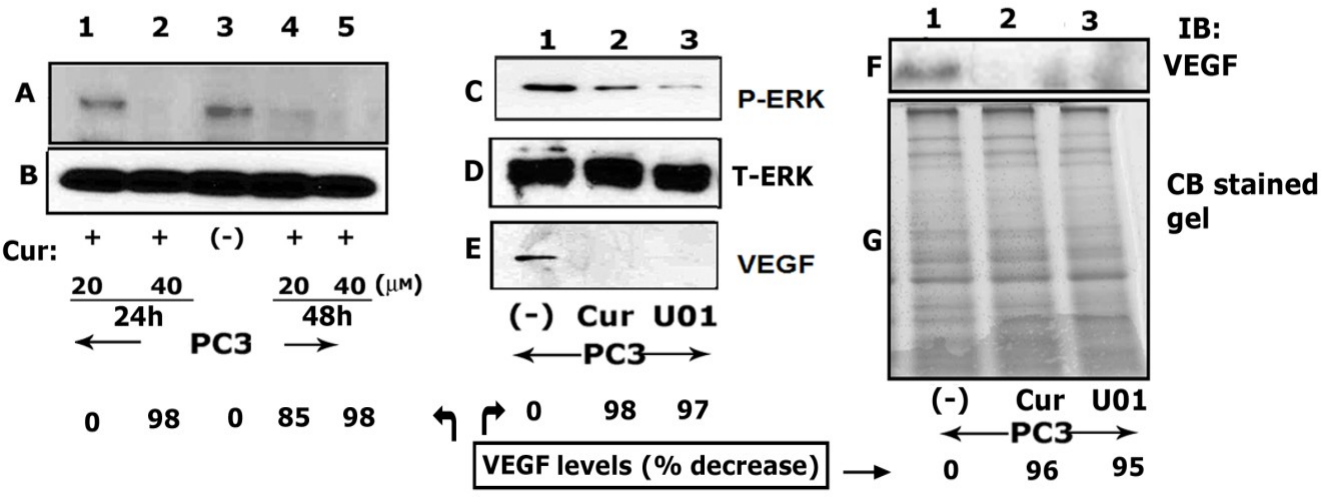
Curcumin reduces ERK phosphorylation and VEGF expression in PC3 cells. (**A**) and (**B**) PC3 cells were treated with 20 and 40 µM curcumin for 24 (lanes 1 and 2) and 48h (lanes 4 and 5). Untreated PC3 cells were indicated as (-) in lane 3. (**C**–**E**) Equal amount of lysate protein (20µg protein) made from untreated (-) and curcumin (lane 2) or MEK inhibitor UO126 (lane 3) treated PC3 cells were immunoblotted with a phospho-specific antibody to ERK1/2 (**C**). Membrane was stripped successively and reprobed with a VEGF antibody (**E**) and a non-phosphorylated ERK1/2 antibody (**D**) to demonstrate equal protein loading. (**F**) and (**G**) To determine the secreted levels of VEGF in PC3 cells treated with curcumin (lane 2) and MEK inhibitor UO126 (lane 3), equal amount of conditioned media protein was used for immunoblotting with an antibody to VEGF (**F**). Untreated (-) PC3 cells were used as controls (lane 1). Coomassie blue staining of a gel ran in parallel was used as a loading control (**G**). Percent decrease of VEGF levels for the representative blot is shown at the bottom of the panels. Results shown are representative of three independent experiments.

Curcumin, a polyphenolic medicinal substance, has been implicated as a suppressor of tumor initiation, promotion, angiogenesis, and metastasis [30,31]. PC3 cells were treated with curcumin for 24 and 48 h (Figure 3A). 

Two different concentrations (20 and 40 µM) of curcumin were used. Changes in the expression of VEGF were determined by immunoblotting analysis. VEGF level in untreated PC3 cells is shown in lane 3 (Figure 3A). Curcumin downregulated the expression of VEGF in a dose- and time-dependent manner (lanes 1, 2, 4 and 5). Since comparable downregulation of VEGF was observed with 40 µM curcumin at 24 and 48 h (A, lanes 2 and 5); we chose to continue our experiments with 40 µM curcumin treatment for 24 h.

### 3.4. Curcumin Inhibits ERK Phosphorylation and VEGF Levels in PC3 Cells

To determine whether MAPK pathway is involved in the expression of VEGF and curcumin affects this pathway, PC3 cells were treated with 40 µM curcumin for 24 h. Phosphorylation of ERK1/2 was determined by immunoblotting analysis (Figure 3C). As a control, a MEK inhibitor (U0126; 10 μM for 24 h) was used to inhibit the phosphorylation of ERK1/2 (Figure 3F, lane 3). The data indicate that curcumin significantly inhibits the phosphorylation of ERK1/2 (Figure 3C,F, lane 2). ERK1/2 phosphorylation is significantly reduced in PC3 cells treated with a MEK inhibitor. Inhibition of ERK phosphorylation reduces cellular (lanes 2 and 3 in panel G) and secreted (lanes 2 and 3 in panel I) levels of VEGF. Immunoblotting of the corresponding non-phosphorylated ERK1/2 (D and H) and a GAPDH antibody (E) was used to demonstrate equal loading. Taken together, the data demonstrate that VEGF expression involves MAPK pathway and curcumin abrogates the expression of VEGF by inhibiting ERK1/2 phosphorylation. 

### 3.5. Inhibition of ERK Phosphorylation Represses VEGF Induced Angiogenesis *in Vitro*

**Figure 4:**
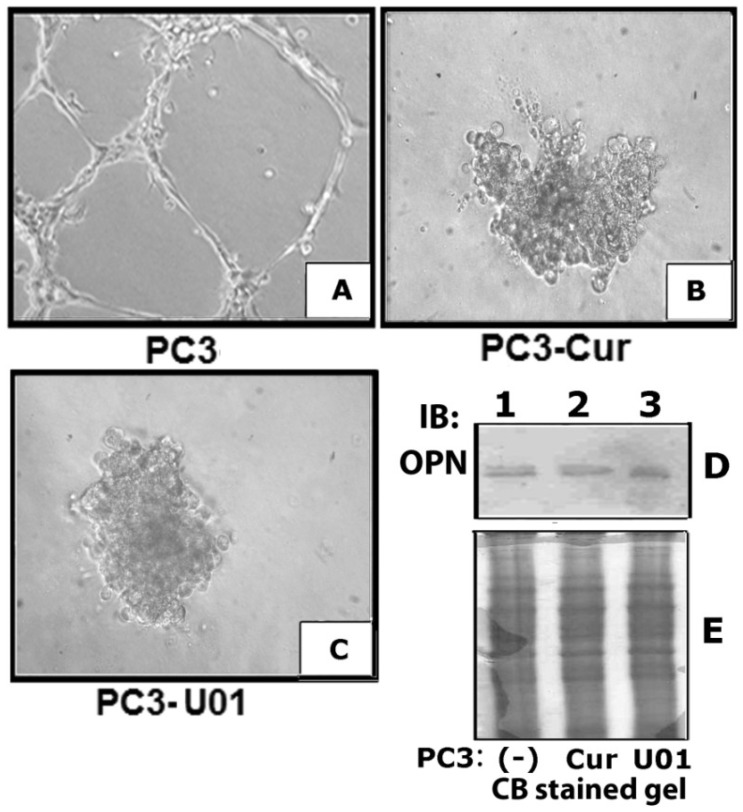
Curcumin and MEK inhibitor reduces microvascular tube formation *in vitro*. (**A**–**C**) Conditioned media collected from untreated (**A**) and curcumin (**B**) or MEK inhibitor UO126 (**C**) treated PC3 cells were used for angiogenesis assay *in vitro* with HMEC-1 cells. Images were captured (×200) with an inverted phase contrast microscope. (**D**) Immunoblotting analysis of secretory levels of OPN in PC3 cells treated with curcumin (lane 2) and MEK inhibitor U0126 (lane 3) is shown. Untreated PC3 cells are indicated as (-) in lane 1. The loading control for the conditioned medium is shown by the use of a Coomassie blue (**E**) staining of a gel ran in parallel. Results shown are representative of three independent experiments.

Conditioned media collected from PC3 cells treated with curcumin (Figure 4B) and MEK inhibitor (C) was used for angiogenesis assay *in vitro* with HMEC-1 cells. HMEC-1 cells incubated with the conditioned medium from PC3 cells were used as controls (A). Consistent with the observation shown in Figure 2A, conditioned medium from PC3 cells induced the formation of tubular sprouts (Figure 4A). Tube formation was significantly inhibited in HMEC-1 cells incubated with conditioned media of curcumin and MEK inhibitor treated PC3 cells (Figure 4B,C). However, proliferation and clustering of HMEC-1 cells was observed under these conditions. The effects of curcumin and MEK inhibitor on the secretory levels of OPN were determined in the conditioned media by immunoblotting analysis. OPN expression and secretion was not affected by these treatments in PC3 cells (D, lane 2 and 3). Clustering and proliferation of HMEC-1 cells may be due to the presence of OPN in the conditioned media. Consistent with the observations by others in breast cancer studies [32]; curcumin inhibits the expression of VEGF in prostate cancer cells and therefore angiogenesis *in vitro*. It exerts its potential inhibitory effect on the OPN/αvβ3-mediated MAPK pathway which may be involved in the expression of VEGF in PC3 cells. 

### 3.6. Matrix Metalloproteinase 9 (MMP9) Plays a Role in the Secretion of VEGF

MMP2 and MMP9 expression has been associated with the progression of neovascular diseases and secretion of VEGF [33,34]. We have observed MMP9 as a principal MMPs in PC3 cells [18,19]. Therefore we generated stable control (PC3/Sc) and MMP9 knockdown (PC3/Si (MMP9) PC3 cell lines as described previously [19]. We examined the association of MMP9 with VEGF expression and secretion using these stable cell lines. 

**Figure 5:**
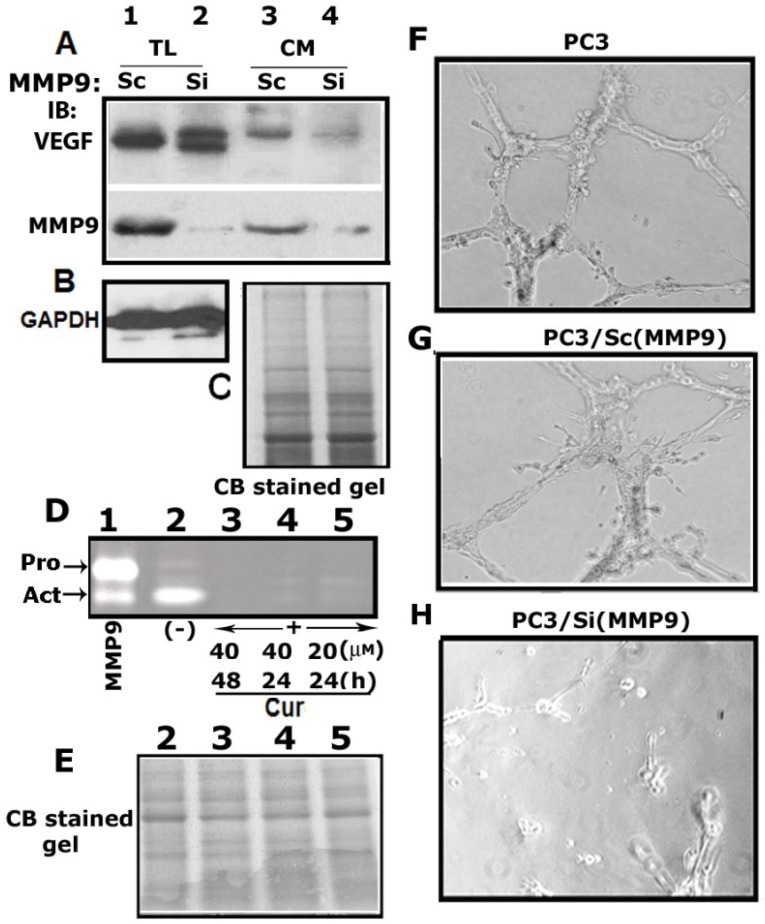
MMP9 plays a role in the secretion of VEGF. (**A**–**C**) Total cellular lysates (TL; lanes 1 and 2) and conditioned media (CM; lanes 3 and 4) made from PC3/Sc (MMP9) (lanes 1 and 3) and PC3/Si (MMP9) (lanes 2 and 4) were immunoblotted with an antibody to VEGF (A, top panel) and MMP9 (A, bottom panel). Equal amount of total cellular lysate protein (20 µg) and conditioned media (20 µg) was used for immunoblotting analysis. Blot in A was stripped sequentially and reprobed with a MMP9 to demonstrate MMP9 levels (**A**, bottom panel) and GAPDH antibody to demonstrate equal protein loading in total cellular lysates (**B**-left panel). Coomassie blue staining of a gel ran in parallel was used as a loading control for conditioned media (**C**). (**D**,**E**) PC3 cells were treated with 20 µM curcumin for 24 h (lane 5) and 40 µM curcumin for 24 (lanes 4) and 48 h (lanes 3). Untreated PC3 cells (-) were used as controls (lane 2). Equal amount of conditioned media (20 µg) was used for zymogram analysis. The activity of a recombinant MMP9 protein containing pro- and active band (indicated by arrows) was used as an identification marker (lane 1). The loading control for the conditioned media used for zymogram analysis is shown by the use of Coomassie blue staining of a gel ran in parallel (**E**). (**F**–**H**) Conditionedmedia from indicated PC3 cells (F-H) were tested for their effect on angiogenesis. HMEC-1 cells were used. Images were captured (×200) with an inverted phase contrast microscope. The results represent one of three separate experiments performed.

As shown in Figure 1 in PC3 cells, we have shown here that control PC3/Sc (MMP9) cells express VEGF 165 (Figure 5A, lane 1). VEGF-165 (denoted as VEGF) is the most common form in tissues and potent angiogenic factor. Expression of VEGF and VEGF165-b (an isoform of VEGF) was found to be significantly elevated in PC3/Si (MMP9) cells (Lane 2). However, only the VEGF isoform is secreted by these cells (Figure 5A, lanes 3 and 4). The secretion is significantly lower in PC3/Si (MMP9) cells (lane 4) than PC3/Sc cells (lane 3). The decrease was found to be > 3 fold in PC3/Si (MMP9) cells as compared with control cells (3.4 ± 0.04; n = 3; ** *p* > 0.001 *vs*. PC3/MMP9 (Sc) cells). These results indicate that MMP9 more potently enhanced the secretion of VEGF in PC3 cells. MMP9 knockdown exert a positive effect on VEGF-165b expression and negative effects on the secretion of both VEGF and VEGF165-b. MMP9 levels in indicated PC3 cells are shown in the bottom panel (A). How the expression and secretion of VEGF-165b is spatially modulated by MMP9 needs further elucidation.

### 3.7. Curcumin Abrogates MMP9 Activity

Curcumin suppressed MMP2 and MMP9 activity in the tumor bearing site of prostate cancer. Therefore, the metastatic nodules *in vivo* were significantly fewer in the curcumin-treated group than untreated group [35]. Conditioned media was used to determine the MMP9 activity by gelatin-zymogram analysis (Figure 5D). As shown by others [35,36], curcumin abrogates MMP9 activity in a dose and time dependent manner (lanes 3–5). A significant decrease in MMP9 activity was observed in PC3 cells treated with 40 µM curcumin for 48 h (lane 3). Targeting MMP9 by curcumin might serve as a potential therapeutic approach for tumor-induced angiogenesis and invasion. 

### 3.8. MMP9 Knockdown Reduces VEGF-Induced Angiogenesis *in Vitro*

MMP9 is implicated in regulating angiogenesis via secretion of VEGF [16]. Conditioned media collected from PC3, PC3/Sc (MMP9), and PC3/Si (MMP9) cells (Figure 5F–H) were used for *in vitro* angiogenesis assay with HMEC-1 cells. The formation of tubular structures (Figure 5F–H) is directly proportional to the amount of VEGF present in the conditioned media of PC3 (Figure 3A,I) and PC3/Sc (MMP9) (Figure 5A) cells. Neither clustering of cells nor elongation of tubular structures was observed in HMEC-1 cells incubated with the conditioned medium of PC3/Si (MMP9) cells (Figure 5H). This may be partly due to a decrease in the proliferation rate. However, these cells displayed migratory phenotype and have a propensity to organize tubular structures (H). MMP9 knockdown in PC3 cells significantly reduced VEGF secretion (Figure 5A, lane 4) and hence tubular extension of HMEC-1 cells *in vitro* (Figure 5H). 

### 3.9. MMP9 Knockdown Increases Angiostatin Secretion by PC3 Cells

**Figure 6:**
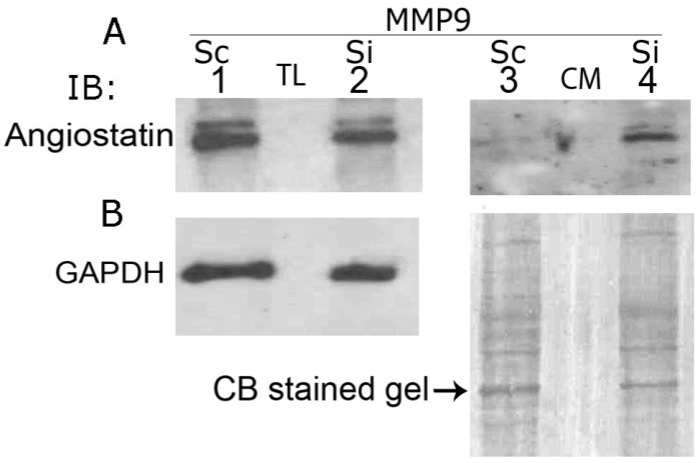
MMP9 knockdown increases secretion of angiostatin in PC3 cells. Total cellular lysates (TL; lanes 1 and 2) and conditioned media (CM; lanes 3 and 4) made from PC3/Sc (MMP9) (lanes 1 and 3) and PC3/Si (MMP9) (lanes 2 and 4) were immunoblotted with an antibody to angiostatin (**A**). Equal amount (20 µg) of protein was used for immunoblotting analysis. Membrane was stripped and reprobed with a GAPDH antibody (**B**, left). GAPDH level was used as a control for loading. Equal protein loading in conditioned media was verified by Coomassie blue (**B**, right) staining of a gel ran in parallel.

Angiostatin is a 38 kDa fragment of plasminogen that selectively inhibits endothelial cell proliferation and angiogenesis. MMPs such as MMP7 and MMP9 regulate the formation of angiostatin fragment(s) from plasminogen [37]. Here we sought to determine whether MMP9 knockdown affect the formation of angiostatin (Figure 6). Immunoblotting analysis indeed has demonstrated the presence of two bands with apparent molecular masses of 42 and 38 kDa angiostatin in total cellular lysates isolated from stable PC3/Sc (MMP9) and PC3/Si (MMP9) cells (lanes 1 and 2). However, angiostatin bands with molecular mass ~42 and 38 kDa were observed only in the conditioned medium of PC3/Si (MMP9) cells (lane 4). Consistently, the 38 kDa band is thicker than 42 kDa in both total cellular lysates (lanes 1 and 2) and conditioned medium (lane 4). An increase in the secretion of 38 kDa angiostatin protein (78 ± 8% *** *p* > 001; n = 3) by PC3/Si (MMP9) cells indicate that MMP9 may play a suppressive role in the secretion of angiostatin from PC3 cells. It seems that MMP9 is not involved in the cleavage of plasminogen and formation of angiostatin in PC3 cells. However, it blocks the release of this vital anti-angiogenic factor and increases the metastatic potential of prostate cancer. 

### 3.10. VEGF Expression Is More in Prostate Adenocarcinoma

**Figure 7:**
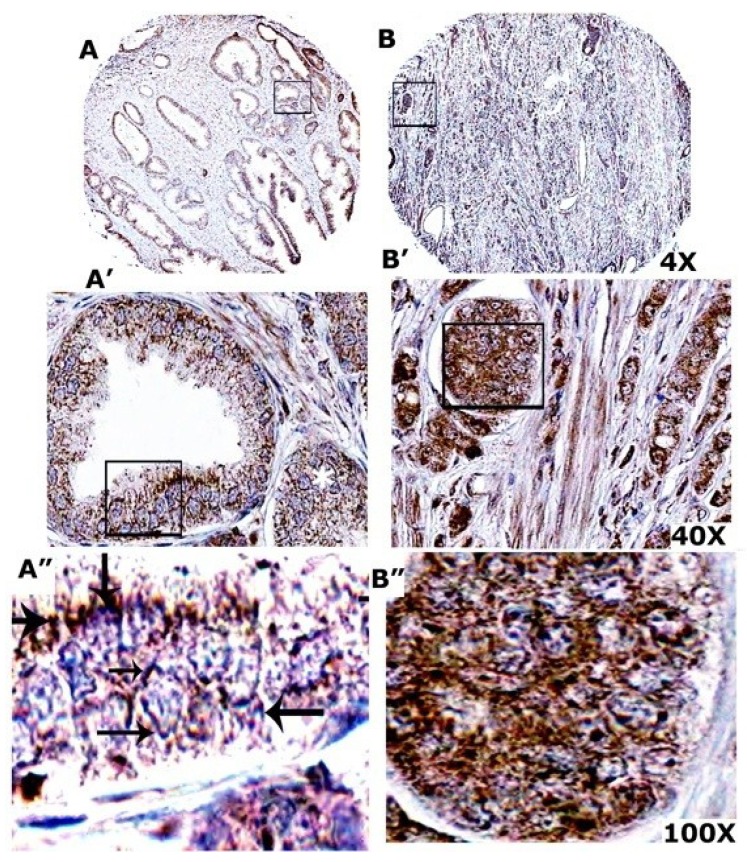
Immunohistochemistry on TMA derived from normal and cancerous prostate tissue. Immunohistochemical staining was performed with an antibody to VEGF in prostate cancer tissue array with adjacent normal prostate tissue. Normal tissue adjacent to prostate cancer is shown in (**A**). Prostate cancer filled lumen adjacent to normal prostate tissue is indicated by a white asterisk. Arrows in (**A''**) point to membrane staining of VEGF in normal cells. Prostate carcinoma at grade 3 is shown in (**B**). Immunostained sections were counterstained with hematoxylin stain (blue). Location of the high magnification regions is indicated by a rectangle field in (**A**), (**A'**), (**B**), and (**B'**). Staining was repeated three times.

Three tissue microarray sections (two PR243a and one PR243 from Biomax) containing six cases of prostate adenocarcinoma and six adjacent normal prostate tissues with duplicate cores for each case were used for immunohistochemistry analysis with an antibody to VEGF (Figure 7). Relative distribution of VEGF in immunostained TMA sections were semi-quantitatively analyzed by two investigators and provided in Table 1. Tissue sections shown in Figure 7A have normal luminal epithelial and hyperplasic prostatic cells with adjacent adenocarcinoma. Normal prostatic luminal epithelial cells and cancer adjacent to normal prostate tissue shows VEGF distribution in the membrane (indicated by arrows in A''). Cancer cells adjacent to normal prostatic epithelial cells appear normal and displayed very similar VEGF distribution to those of normal luminal epithelial cells (A'). Sparse staining was observed in the cytoplasm (A' and A''). VEGF staining is considerably lower in normal prostatic tissue (A–A'') than prostatic carcinoma sections (B–B''). Sections with adenocarcinoma at stage II and IV as well as adenocarcinoma with necrosis displayed cells with significant VEGF staining in the cytoplasm (B' and B''; Table 1) as compared with cancer adjacent to normal prostate tissue and normal luminal epithelial cells. This is consistent with the observations shown by others in prostatic adenocarcinoma [38]. Immunohistochemistry analysis corroborates the findings of the immunoblotting analysis (Figure 1G). 

**Table 1:** Expression of VEGF in prostatic carcinoma and cancer adjacent to normal prostate tissue sections. Prostatic carcinoma and normal tissue microarray containing 12 cases/24 cores was used. Immunohistochemistry was performed with an antibody VEGF. Staining was repeated thrice with two different microarrays (PR243 and 243a; Biomax). ** *p* < 0.01 and *** *p* < 0.001 staining intensity *vs.* normal cells.

Grade	Grade/Stage/# of cores	Cells	VEGF
Normal prostatic epithelial cells and PCa to these cells	(-)/n = 16	Cancer cells appear normal	Normal cells = 28.0 ± 13%
			PCa = 33 ± 12%
			Stromal cells < 10%
Adenocarcinoma (Type: Malignant)	2/IV/n = 16	Cells appear slightly different than normal; moderately differentiated	PCa = 68.7 ± 18% **
			Stromal cells ~6–8%
Adenocarcinoma with necrosis (Type:Malignant)	3/II/n = 8	Cells appear abnormal; poorly differentiated; stroma is less	PCa = 78 ± 22% ***
			Stromal cells ~5–8%
Adenocarcinoma (Type:Malignant)	3/II/n = 8	Cells appear abnormal; poorly differentiated; stroma is less.	PCa = 82 ± 28% ***
	3/IV/n = 16		Stromal cells ~4–6%

## 4. Discussion

Osteopontin has been discovered as the leading candidate clinical marker derived from a screen of approximately 12,000 named genes [39]. We demonstrated previously that OPN expression increases prostate cancer progression through the formation of invadopodia-like structures through integrin αvβ3 signaling pathway [25]. OPN has been related to the development and progression of tumor through its angiogenic potential [32,40]. The present study was undertaken to determine the role of OPN in VEGF expression and angiogenesis. The study tested whether OPN induced MAPK pathway has a role in the expression of VEGF and whether MMP9 has a role in the expression and/or secretion of VEGF. ERK inhibitor has been shown to regulate the expression of VEGF [41,42]. The comparable effects of curcumin and an ERK inhibitor on ERK phosphorylation and VEGF expression has been evaluated in this paper. Curcumin has been shown to block MMP2 and MMP9 activity [36], (reviewed in [43]). We have determined the anti-angiogenic effects of curcumin by comparing its effect with an ERK inhibitor and PC3 cells knockdown of MMP9. 

**Figure S1:**
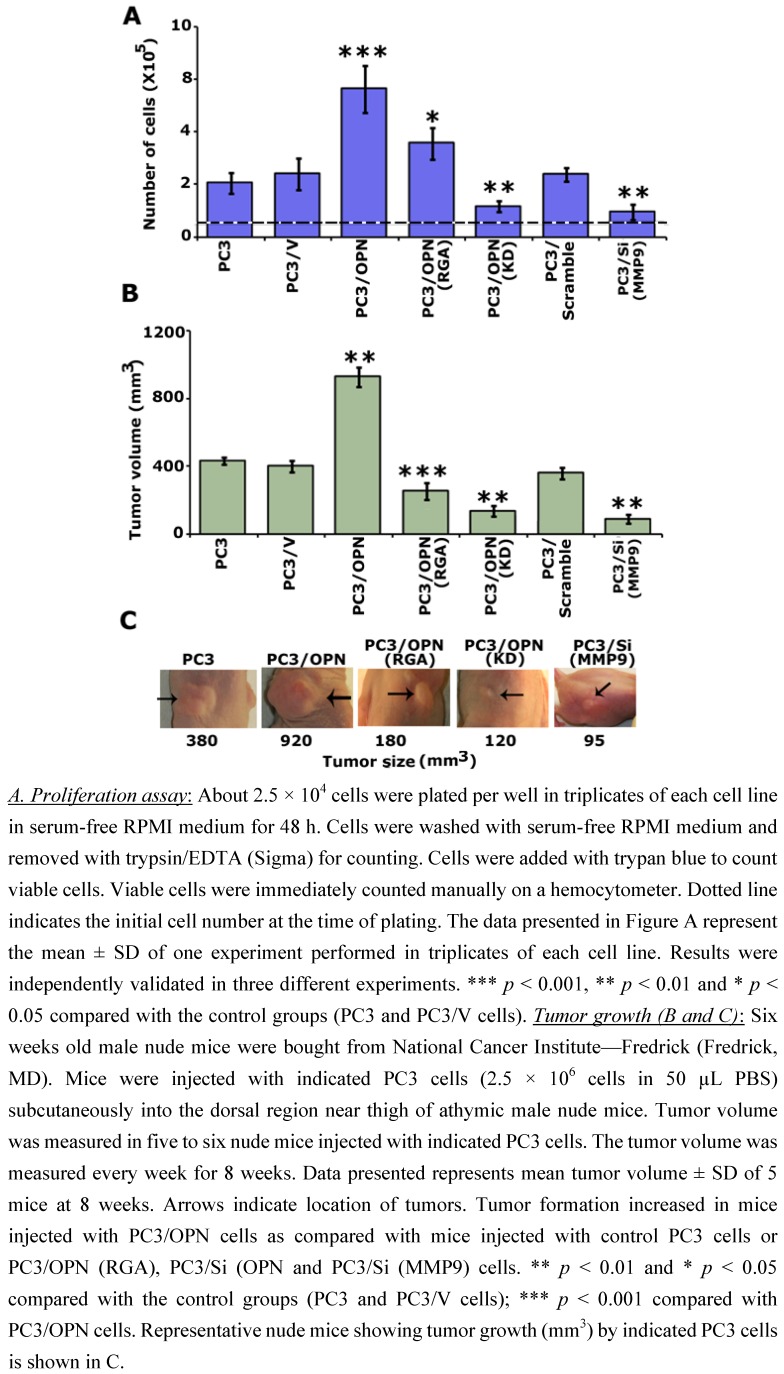
OPN over-expression increases proliferation of PC3 cells and the ability of these cells to form big tumor in nude mice. Cells at log phase of growth were used for proliferation assay in vitro in 48 well plates (**A**) and tumor growth experiment *in vivo* in nude mice (**B** and **C**).

We confirmed that OPN over-expression in PC3 cell line enhanced proliferation (Supplemental Figure S1) of PC3 cells and increased the tumorigenecity. The tumor size corresponds with expression levels of OPN. PC3/KD (OPN) and PC3/OPN (RGA) cells produced very small tumors (Supplemental Figure S1). Using tissue microarray analysis and lysates from prostatic tumor cells, we have observed that expression of OPN (Supplemental Figure S3) and VEGF was more pronounced in prostate cancer as compared with benign or normal prostatic tissue. Rates of VEGF expression were also shown to be significantly lower in benign prostatic *vs.* prostate cancer specimens [38]. It is well established that VEGF plays an important role in the maintenance of angiogenesis and has systemic effects at secondary sites on tumor growth [44,45]. Several stimuli regulated the expression and secretion of VEGF. We postulated that OPN-mediated signaling may regulate the expression and secretion of VEGF in prostate cancer cells.

In endothelial cells, OPN has been shown to induce angiogenesis through activation of ERK-mediated pathways with VEGF acting as a positive feedback signal [46]. MAPK signaling pathway which involves ERK1/2 seems to be responsible for VEGF secretion in multiple myeloma and breast cancer cells [47]. The down-modulation of ERK1/2 activity with an inhibitor reduces myeloma induced angiogenesis by inhibiting VEGF secretion [41]. In this study we found that MEK inhibitor suppresses ERK1/2 phosphorylation as well as VEGF expression and secretion in PC3 cells. Curcumin has been shown to suppress multiple signaling pathways and inhibit cell proliferation, invasion, metastasis, and angiogenesis (reviewed in [43]). However, its effect on VEGF expression by MAPK pathway and MMP9-mediated secretion of VEGF needs further elucidation. Recently, we have reported that activation of c-Raf-ERK cascade may promote cell cycle arrest in prostate cancer cells and OPN signaling has a role in the anti-apoptotic mechanism [48]. Curcumin has been shown to have anti-tumor effects in prostate cancer [32,35]. MAPK is shown to be one of the targets of curcumin in VEGF-mediated angiogenesis in human intestinal microvascular endothelial cells [49]. Our results in the present study also suggest that ERK plays a role in VEGF expression. Curcumin has comparable inhibitory effect as ERK inhibitor in the expression of VEGF. 

**Figure S2:**
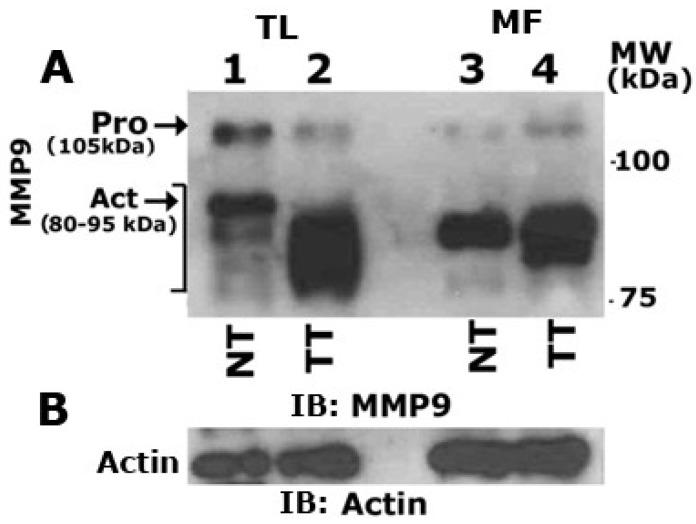
Expression levels of MMP9 in human prostate normal (NT) and tumor (TT) tissue. Immunoblotting analysis. Total cellular lysate (TL; lanes 1 and 2) and membrane fraction (MF; lanes 3 and 4) of the lysate from normal (NT) and tumor (TT) prostatic tissue were immunoblotted with antibodies to MMP9 (**A**). Equal loading of membrane protein was shown by immunoblotting with an actin antibody (**B**) after stripping the blot. The results represent one of three separate experiments performed. As shown in PC3 cells [18], an increase in the levels of active form of MMP9 was observed in total cellular lysates and membrane fraction (**A**, lanes 2 and 4)of the prostate tumor tissue (TT) as compared with normal tissue (lanes 1 and 3 in **A**). The active form of MMP9 is more in tumor tissue (lanes 2 and 3).

**Figure S3:**
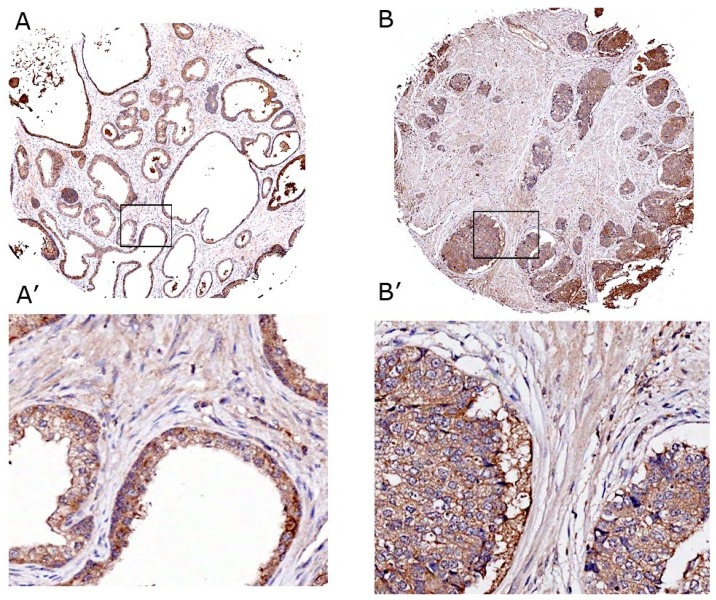
Immunohistochemistry on tissue microarray sections containing normal tissue adjacent to adenocarcinoma and adenocarcinoma at grades 1 to 3. Immunohistochemical staining was performed with an antibody to osteopontin. Normal tissue adjacent to prostate cancer (**A**) and adenocarcinoma at grade 2–3 (**B**) are shown. Sections were immunostained (brown) as described in the Methods section. Immunostained sections were counterstained with hematoxylin stain (blue). Location of the high magnification regions are shown by a rectangle field in (**A**) and (**B**) Magnification is 50× in A and B and 200× in (**A'**) and (**B'**). The staining was repeated two times with similar results.Staining was observed in stromal cells, normal prostatic tissue, and adenocarcinoma in lumen. Cytosolic staining was observed in these cells. Adenocarcinoma at grade 2–3 is shown in (**B**) and (**B'**). Prostatic adenocarcinoma sections showed multiple small foci of tumor cells. Cells appear slightly different than normal. One of the foci is showed in higher magnification (**A'** and **B'**). An increase in number of cells in the foci of adenocarcinoma corresponds with intense staining of cells (**B'**). A part of the reason for more OPN in the lysates from tumor tissue (Figure 1 in the main document) may be due to an increase in the number of cancer cells in the foci which express more OPN.

MMP9 is the principle MMP although MMP2 is also expressed in PC3 cells. Data presented here and previously, demonstrated membrane localization of pro- and active forms of MMP9 and secretion of only active form of MMP9 in PC3 cells [18]. OPN over-expression increased the activity of cell surface and secreted MMP9 [18]. We have previously shown an increase in the levels of MMP9 in tissue microarray sections containing adenocarcinoma at stages 2 to 4 [23]. Consistent with the observations shown in PC3 cells [18], high expression of active form of MMP9 was observed in prostatic adenocarcinoma tissue lysates (Supplemental Figure S2). MMP9 seems a promising target for preventing angiogenesis, invasion, and metastasis in cancer patients. Our results are in agreement with previous reports of inhibition of MMPs activity by curcumin (reviewed in [43,50,51]). Curcumin blocks MMP9 activity and secretion of VEGF in addition to its effect on the reduced expression of VEGF through suppressing ERK1/2 phosphorylation in PC3 cells. However, expression and secretion of OPN is unaffected by MEK inhibitor or curcumin. Therefore, OPN present in the conditioned medium of PC3 cells treated with MEK inhibitor or curcumin stimulated proliferation and clustering of HMEC-1 cells through cell-cell adhesion but not cell migration and tube formation. 

MMP9 knockdown PC3 cells corroborate the relevance of MMP9 in angiogenesis through its role in the secretion of VEGF. VEGF 165 (indicated as VEGF) and VEGF165-b are expressed in PC3/Si (MMP9) cells. VEGF165 was not observed in PC3 cells expressing scrambled RNAi or control PC3 cells. It is not known whether the secreted or membrane bound MMP9 controls VEGF release. However, studies using PC3/Si (MMP9) cells evidently suggest a role for MMP9 in the secretion of VEGF. Induction of MMP9 activity may be a likely mechanism by which VEGF is released from prostate cancer.

The intriguing observation in the present study is that while the total cellular levels of angiostatin remains the same in PC3/Sc (MMP9) and PC3/Si (MMP9) cells, an increase in the release of angiostatin was observed in PC3/Si (MMP9) cells. As MMP9 knockdown increases the release of angiostatin, we cannot exclude the fact that MMP9 is a potential blocker of angiostatin release from PC3 cells. It is possible MMP9 may increase the angiogenic potential via suppression of secretion of angiostatin by unknown mechanism. It would be interesting to find out how MMP9 suppresses the release of angiostatin. Angiostatin produced from plasminogen by MMP7 and MMP9 has been shown to have biological activities such as inhibition of endothelial cell proliferation, angiogenesis, tumor growth and metastasis [52]. It is not known whether MMP7 is involved in the release of angiostatin in the absence of MMP9. 

## 5. Concluding Remarks and Future Directions

VEGF expression and secretion correlates well with the levels of OPN in PC3 cells. OPN/integrin αvβ3 signaling involving MAPK pathway plays a role in the expression of OPN. ERK inhibitor and curcumin has comparable effects on the inhibition of VEGF expression. However, curcumin also has an inhibitory effect on the activation of MMP9. Experiments with PC3 cells knockdown of MMP9 and PC3 cells treated with curcumin show a positive feedback regulation between MMP9 activity and VEGF secretion. MMP9 appears to have negative regulatory role in the secretion of VEGF165b and angiostatin. VEGF-165b is implicated as anti-angiogenic factor [9]. Angiostatin has been implicated as a potent inhibitor of angiogenesis and reported to be one of the factors which suppresses the growth of secondary tumors in mice bearing previous tumors. However, the mechanism by which angiostatin suppresses the growth has not been proven [53,54,55]. Understanding the different roles of MMP-9 should allow the development of better therapeutic strategies in the treatment of cancer. This interesting observation stimulated our interest in the identification of the mechanism that induces the release of angiostatin from PC3/Si (MMP9) cells. 

## Abbreviations

OPN: Osteopontin
VEGF: vascular endothelial growth factor
MAPK: mitogen-activated protein kinase
ERK1/2: extracellular signal-regulated kinases 1 and 2
MEK1 inhibitor: MAP/ERK kinase 1 inhibitor
MMPs: matrix metalloproteinases
MMP9: matrix metalloproteinase 9
integrin αvβ3: vitronectin receptor
RGD: amino acid sequences such as, Arginine(R), Glycine (G) and Aspartic acid (D). It is also known as integrin binding motif
RGA: amino acid sequences such as Arginine (R), Glycine (G) and Alanine (A)
PC3/OPN: PC3 cells over expressing OPN
PC3/OPN (RGA): PC3 cells over-expressing mutated OPN in integrin binding motif (RGDΔRGA)
PC3/OPN (KD): PC3 cells knockdown of osteopontin
PI3-K: phosphatidylinositol 3-kinases
HMEC-1 cells: human microvascular endothelial cells-1
